# Feasibility and acceptability of follow-up for prostate cancer in primary care: a pilot study

**DOI:** 10.3399/bjgpopen18X101616

**Published:** 2018-12-12

**Authors:** Marianne Heins, François Schellevis, Mirjam Schotman, Bart van Bezooijen, Ismene Tchaoussoglou, Mirjam van der Waart, Lilan Veldhuis, Sandra van Dulmen, Gé Donker, Joke Korevaar

**Affiliations:** 1 Researcher, Department of General Practice, Netherlands Institute for Health Services Research (NIVEL), Utrecht, The Netherlands; 2 Professor, Department of General Practice & Elderly Care Medicine, Amsterdam Public Health Research Institute, VU University Medical Centre, Amsterdam, The Netherlands; 3 Senior Researcher, Department of General Practice, Netherlands Institute for Health Services Research (NIVEL), Utrecht, The Netherlands; 4 Urologist, Department of Urology, Meander Medical Centre, Amersfoort, The Netherlands; 5 Urologist, Department of Urology, Meander Medical Centre, Amersfoort, The Netherlands; 6 Medical Coordinator, MCC Eemland, Amersfoort, The Netherlands; 7 GP, Lepelaar GP Practice, Leusden, The Netherlands; 8 GP, Lepelaar GP Practice, Leusden, The Netherlands; 9 GP, Veldhuis/Blom GP Practice, Amersfoort, The Netherlands; 10 Professor, Department of Primary and Community Care, Radboud University Medical Center, Radboud Institute for Health Sciences, Nijmegen, The Netherlands; 11 Professor, Faculty of Health and Social Sciences, University of South-Eastern Norway, Drammen, Norway; 12 Programme Coordinator, Department of Communication in Health Care, Netherlands Institute for Health Services Research (NIVEL), Utrecht, The Netherlands; 13 Senior Researcher, Department of General Practice, Netherlands Institute for Health Services Research (NIVEL), Utrecht, The Netherlands; 14 Programme Coordinator, Department of General Practice, Netherlands Institute for Health Services Research (NIVEL), Utrecht, The Netherlands

**Keywords:** primary health care, general practice, neoplasms, prostatic neoplasms, aftercare, health services

## Abstract

**Background:**

The number of patients with prostate cancer is increasing, which puts additional pressure on health care. GP-led follow up may help reduce costs, travel time for patients, and workload for urologists and improve continuity of care.

**Aim:**

To test the feasibility and acceptability of a new clinical pathway for GP-led prostate cancer follow-up.

**Design & setting:**

A feasibility pilot study was performed in cooperation with six GP practices in the Dutch region of Amersfoort.

**Method:**

The study included 20 patients with prostate cancer in a stable phase, who were aged ≥65 years and with comorbidity. Follow-up for prostate cancer was transferred to the GP for one year. Participating GPs and urologists jointly developed a protocol. Patient satisfaction was measured at 0 and 12 months with the ‘personalised care’ subscale of the Consumer Quality (CQ) index 'general practice care'. Next, patients, GPs, and urologists were interviewed about their experiences. The clinical pathway was considered successful if no patients were referred back to the urologist, except for an increase in prostate-specific antigen (PSA), and if the majority of patients and participating urologists and GPs were satisfied.

**Results:**

Of the 20 patients included in the study, three were referred back to the urologist because of increasing PSA levels and one died (unrelated to prostate cancer). Most patients (73%) were satisfied with the transfer of care, indicated by a score of ≥3 on the ‘personalised care’ subscale. GPs and urologists had confidence in the ability of GPs to provide follow-up care and preferred to continue this.

**Conclusion:**

The new clinical pathway was successful, warranting a larger study to provide evidence for the (cost-)effectiveness of GP-led prostate cancer follow-up.

## How this fits in

The number of people surviving prostate cancer is increasing, which puts an extra demand on health care. Follow-up by the GP may help to reduce workload for urologists, travel time for patients, and improve continuity of care. This pilot study has found that GP-led follow-up for older patients with prostate cancer in a stable phase is feasible and acceptable for patients, GPs, and urologists. Therefore, a larger study to provide evidence for the (cost-)effectiveness of GP-led prostate cancer follow-up is warranted.

## Introduction

The number of patients with prostate cancer is high and is set to increase even further.^1,2^ This growing patient group will put an increasing demand on health care, as most patients receive hospital follow-up,^3–5^ many of them are older ^2^ and many have one or more chronic diseases besides prostate cancer.^6^ This calls for evaluation of the organisation of care for these patients.^7^

Responsibility for the care of patients with prostate cancer is now often shared between several care providers; urologists provide prostate cancer-specific care, while other physicians, for example, provide care for chronic diseases. Consequently, care providers have to inform each other about test results and medication changes, and patients have to express their wishes and preferences to several care providers. In addition, travelling to these care providers may be challenging for older patients.

When prostate cancer is in a stable phase and patients have comorbid conditions, a patient-centred approach is needed rather than a disease-oriented approach. In countries with a strong primary care system, this could be achieved by increasing the role of the GP in the follow-up of prostate cancer.

This has several potential benefits; it is more patient friendly, for example, as consultations for follow-up of prostate cancer and comorbid conditions may be combined, and the GP practice is often nearby. Costs will probably be lower and workload for urologists will be reduced. As the GP has a complete overview of the patient’s medical record, it may improve continuity and quality of care.

Several trials in other cancer types provided evidence for these benefits;^8^ however, there are also some potential obstacles. Patients and urologists may not view the GP as knowledgeable on cancer and prefer follow-up at the hospital.^7,9^ GPs may feel that they lack knowledge about cancer and follow-up requirements, and may face a high workload and lack of financial reward for additional tasks.^9^

To assess acceptability and feasibility of GP-led follow-up for prostate cancer, a pilot study was performed. In the study, a limited number of older patients with prostate cancer and comorbidity were entirely transferred to the GP for follow-up for 1 year.

## Method

### Study design

A new clinical pathway for older patients with prostate cancer and comorbidity was implemented and evaluated. This pilot study was performed in the urology department of Meander MC Amersfoort, in cooperation with six GP practices (*n *= 14 GPs, one participating in each practice) from the same region. Participating patients received care according to the clinical pathway and were followed for 12 months.

### Clinical pathway

After receiving the referral letter from the urologist, the GP met patients at the times established in the clinical pathway protocol (further information available from the authors on request) and at check-ups for their chronic disease. Patients could always contact their GP in between these appointments if needed.

GPs asked about symptoms, ordered PSA measurements, and discussed results with patients. If needed, GPs could consult a urologist at short notice and refer patients to them. GPs could also arrange a video consultation with the urologist (with or without the patient). It was hypothesised that this could be used to discuss problems with the GP, urologist, and patient together, which could support shared decision-making.

### Development of the clinical pathway protocol

Two participating urologists and GPs developed the protocol for the clinical pathway, which was based on existing national guidelines and endorsed by all participating urologists and GPs (further information available from the authors on request) It contained background information, detailed inclusion criteria, a follow-up schedule, symptoms to ask about (specified by treatment group, for example, new urinary symptoms, or erectile dysfunction), and criteria for referral to the urologist. During the study, the protocol was evaluated and no changes were needed.

### Patient inclusion

Patients with prostate cancer receiving follow-up at the participating hospital and listed with a participating GP practice were screened for eligibility. The inclusion criteria were as follows:

- prostate cancer in a stable phase (defined in the protocol);
- age ≥65 years; and
- ≥1 comorbid chronic disease.^10^

The exclusion criteria were as follows:

- active medical treatment by urologist;
- planned discontinuation of follow-up within 1 year;
- follow-up by GP not medically safe (according to the urologist); and
- patients under ‘active surveillance’.

### Outcome

#### Primary outcome

The primary outcome of this study was a successful clinical pathway, according to three criteria discussed below.

##### No referral back to the urologist unless indicated

A clinical pathway could only be deemed successful if GPs and patients trust the GP's expertise sufficiently to refrain from referring patients back to the urologist. An exception was, of course, in cases of a rise in PSA levels or symptoms suggesting disease progression.

##### The majority of the patients are satisfied

Patient satisfaction was measured 12 months after inclusion using the ‘tailored care’ subscale of the CQ-index ‘general practice care’.^11 ^ This subscale consists of nine questions, and scores range from 1–4. The average score in the Dutch population is 3.2. The reliability of the subscale was shown to be good (Cronbach’s alpha 0.88).^11^ When two-thirds of the patients had a score of ≥﻿3,the clinical pathway was judged to be successful. In addition, one of the researchers interviewed six random patients at the end of the study about their experiences. Questions regarding satisfaction, logistical and/or practical problems, and trust in the GP were defined beforehand by two of the researchers. Answers were written down and analysed by the same researchers.

##### The participating GPs and urologists are satisfied

One of the researchers interviewed all participating urologists and GPs at the end of the study. During the interviews, they specifically asked predefined questions regarding satisfaction, logistical and/or practical problems, uncertainties, and communication. When all GPs and urologists were satisfied and were willing to continue this type of care, the clinical pathway was judged to be successful. If some of the (relatively motivated) urologists and GPs participating in this feasibility study found the pathway unsuccessful, this would be an indication that many urologists or GPs would not be willing to participate if the pathway would be implemented on a larger scale.

#### Secondary outcomes

##### Quality of care from the patient’s perspective

Quality of care from the patient’s perspective was measured 12 months after inclusion with questions derived from the CQ-index ‘care for cancer’.

##### Health-related quality of life

Health-related quality of life was measured 12 months after inclusion with the ‘general health’ subscale of the RAND-36, consisting of five items, measured on a 5-point Likert-scale. Scores range between 0–100, and higher scores indicate better quality of life.^12^

Prostate cancer-specific quality of life was measured 12 months after inclusion with the Dutch translation of the Expanded Prostate Cancer Index Composite,^13^ consisting of 26 questions on two domains: urinary problems and bowel problems.

Fatigue was measured 12 months after inclusion with the 'fatigue' subscale of the 'checklist individual strength'. Scores range from 8–56 and a score ≥35 indicates severe fatigue.^14^

##### Healthcare use

Contacts with care providers during the study were monitored to indicate: (1) involvement of different providers in the care for patients; and (2) patient safety, as complications will most likely lead to additional healthcare use. Patients were asked whether they had contacts in the past 6 months with several types of healthcare providers (for example, GPs, practice nurses, urologists, and other medical specialists) at 6 months and 12 months after inclusion. Data on contacts with specialists and nurses from Meander Medical Center were extracted from the electronic medical records.

##### Use and acceptance of video consultations

The authors monitored the use of video consultations and discussed them during the interviews with GPs and urologists using the unified theory of acceptance and use of technology (UTAUT) model, which consists of the following components: (1) performance expectancy; (2) effort expectancy; (3) social influence; and (4) facilitating conditions.^15^

### Statistical analysis

As this was a pilot study of a clinical pathway that had yet to be tested, the number of subjects included in the study was limited to 20. This was deemed large enough to assess feasibility and acceptability. Given this limited number, only descriptive statistics were calculated. Analyses were performed using Stata/SE (version 13.1).

## Results

### Participants

Fifty-five patients with prostate cancer from the participating hospital were listed with a participating GP practice. Twenty-seven (49%) did not fulfil eligibility criteria, mainly because they were under active treatment (*n *= 10), had progressive disease (*n *= 8), or were no longer receiving follow-up (*n *= 3). Twenty-one of the 28 patients who fulfilled eligibility criteria (75%) agreed to participate. One dropped out before care was transferred to the GP because of a rise in PSA levels, so 20 patients were ultimately included (Table 1).

**Table 1:** Patient characteristics at inclusion (*n *= 20)

Characteristic	*n* (%)
**Mean age, SD**	77.9 (6.1)
**Time after diagnosis^a^**	
<5 years	10 (50)
5–10 years	7 (35)
>10 years	3 (15)
**Treatment**	
Prostatectomy	8 (40)
Radiotherapy	10 (50)
Hormonal therapy	5 (25)
Watchful waiting	3 (15)
General health^b^	51.1 (20.7)
**GP visits in past 12 months**	
1	1 (5)
2–4	9 (45)
5–9	8 (40)
≥﻿10	2 (10)

^a^At inclusion. ^b^Measured 12 months after inclusion with the ‘general health’ subscale of the RAND-36: five items, measured on a 5-point Likert-scale.

SD = standard deviation.

### Criteria for a successful clinical pathway

#### Referral back to the urologist

During the study, three patients were referred back to the urologist, according to the protocol, because of increasing PSA levels. They were aged 68 years, 73 years, and 81 years; were 4, 5, and 9 years post-diagnosis; and had received prostatectomy, radiotherapy followed by hormonal therapy, and watchful waiting. One patient died because of a disease unrelated to prostate cancer.

#### Patient satisfaction

The mean score on the ‘tailored care’ subscale of the CQ-index 'general practice care' after 12 months was 3.3 (SD 0.6). Fourteen patients (74%) had a mean score of ≥3, indicating high satisfaction, two (11%) had a mean score <3, indicating low patient satisfaction (Table 2).

**Table 2:** Outcome after 12 months (*n *= 19)^a^

Outcome	*n* (%)
**Patient satisfaction**	
Satisfied (score ≥3)	14 (74)
Unsatisfied (score <3)	2 (11)
Too many items rated as ‘not applicable’	3 (16)
**General health^b^**	52.4 (18.4)
**Prostate-related symptoms**	
Severe incontinence	9 (47)
Severe bowel problems	2 (11)
Severe fatigue	7 (37)
**Healthcare providers visited in past 12 months**	
GP	19 (100)
Practice nurse	7 (37)
Specialist (excluding urologist)	6 (32)
Physiotherapist	5 (26)
Urologist^c^	4 (21)

^a^One patient died during the observation period, so no outcome was recorded. ^b^Measured 12 months after inclusion with the ‘general health’ subscale of the RAND-36: five items, measured on a 5-point Likert-scale. ^c^Three patients were referred back to the urologist and one saw the urologist because he inadvertently received a letter that he had missed his follow-up visit at the hospital.

Six patients were interviewed about their experiences. They were all satisfied with the follow-up they had received from their GP. Most indicated that it was very similar to the care they had received from their urologist. All trusted the GP’s ability to perform this type of care and if they had questions the GP was able to answer them. Patients experienced no problems with access to GP care or other practical problems. No longer seeing the urologist was not perceived as a problem. One patient had been referred back to the urologist, and was positive about the pathway and the quick referral to the urologist. Nearly all (apart from one patient) preferred to continue follow-up with the GP as the GP practice was nearby, they knew the GP well, and it was easy to discuss other problems. Combining consultations for chronic diseases was only mentioned by one patient. The patient who preferred to consult the urologist explained that he experienced the urologist as being more cordial and he performed physical examination.

#### Satisfaction of urologists and GPs

The participating GPs were satisfied with the clinical pathway. They were confident providing this type of care, as the protocol provided clear criteria for referral to the urologist. Some perceived scheduling and keeping track of follow-up appointments as challenging; as they only had a few patients participating, it was not integrated into their routine procedures. They could always reach a urologist if necessary. Referral letters at the start of the study were sometimes delayed or contained only very basic information. GPs were willing to continue this type of care, provided there were clear arrangements with the urologist about referral and responsibilities.

The two participating urologists were satisfied with the clinical pathway. They already had confidence in the ability of GPs to provide this type of care before the study. Collaboration with the participating GPs was good. They were only contacted a few times, due to an increase in PSA levels. They were willing to continue this clinical pathway.

#### Secondary outcome measures

##### Quality of care

**Figure 1.**
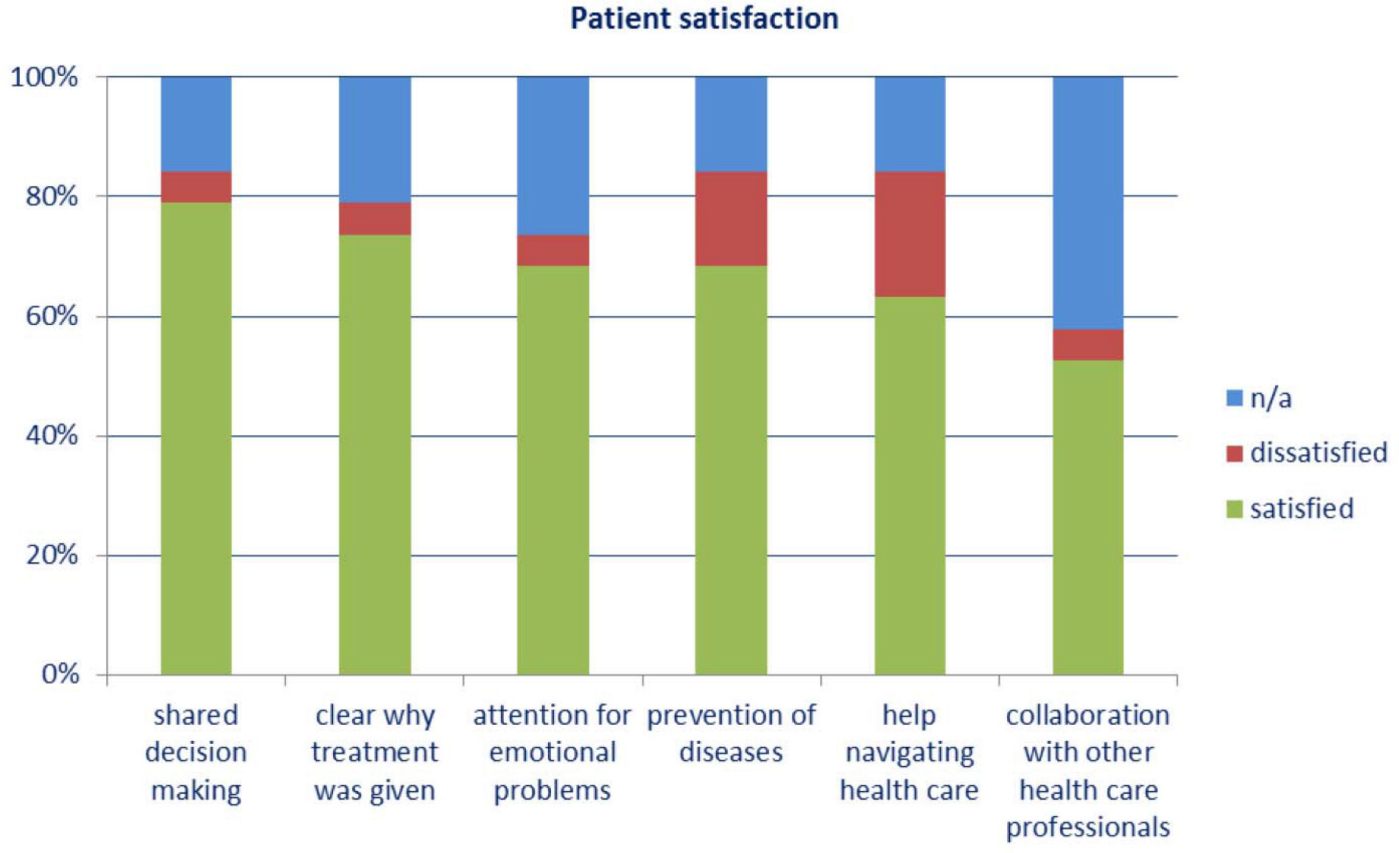
Rating of different components of quality of care n/a= rated as not applicable.

Most patients were satisfied with the quality of care, although some items were rated ‘not applicable’ relatively often (Figure 1).

##### Health-related quality of life

General health did not change during the study period; mean scores changed from 51.1 (SD 21) to 52.4 (SD 18), as shown in Table 2. At the end of the study, many patients still experienced severe urinary incontinence and severe fatigue (Table 2).

##### Healthcare use

During the study patients most often visited their GP, a practice nurse, or a specialist other than a urologist (see Table 2).

##### Video consultations

Video consultations were not used during the study period. GPs expected they would be just as useful as a telephone call (performance expectancy), but would require more effort (effort expectancy). GPs did not feel the need for a video consultation together with the patient, which was their hypothetical added value.

## Discussion

### Summary

The new clinical pathway for older patients with prostate cancer and comorbidity was successful as no patients were referred back to the urologist unless indicated by the protocol, and the majority of participating patients, urologists, and GPs were satisfied.

### Strengths and limitations

Owing to its small sample size, this pilot study cannot provide evidence for (cost-)effectiveness. However, previous studies found that GP-led follow-up for patients with breast and colorectal cancer leads to a similar quality of care at lower costs. Besides, the heterogeneous sample enabled the authors to see whether the pathway was not just feasible for patients with diverse characteristics (for example, short time after diagnosis, older age, and certain treatment).

In this study care was given by dedicated and motivated GPs. If the pathway is implemented on a larger scale, GPs may be more hesitant because of the barriers mentioned in the introduction. Finally, follow-up was only 1 year, so it is not known how many patients returned to their urologist after this period. However, at the end of the pilot most patients preferred to continue receiving follow-up care from their GP.

### Comparison with existing literature

One earlier study found that involving the GP in prostate cancer follow-up produced clinically comparable outcomes to standard hospital follow-up.^16^ However, in that study, follow-up visits were alternated between the urologist and the GP. The present study showed that complete transfer of follow-up to the GP is feasible and acceptable for older patients with comorbidity.

### Implications for research and practice

Some important elements of the clinical pathway were revealed in this study. First, the clear and concise protocol, developed jointly by urologists and GPs, which was sufficient for GPs to perform this type of care. It also reassured them by showing at a glance the (relatively simple) tasks they had to perform.

A second important element was familiarity and short communication lines between GPs and urologists, which fostered mutual trust. Providing video consultations as an extra means of communication and to enable consultation with the GP, urologist, and patient, could be omitted in the future.

There were also elements that could be improved. Information exchange around the initial referral to the GP was sometimes challenging. Extant studies also indicate that GPs do not always receive sufficient and timely information from specialists.^17–20^ The referral process should, therefore, receive more attention in the protocol.

The results warrant a larger study to provide evidence for the (cost-)effectiveness of the clinical pathway. In this trial the target population may well be broadened to younger patients or those without comorbidity, as results do not indicate that the pathway is exclusively suitable for older patients, or that consultations for prostate cancer and chronic diseases are often combined. In addition, seeing more patients would facilitate internalisation of the protocol and implementation in practice management procedures.

However, seeing more patients would also raise the workload for GPs, which is already increasing owing to other tasks that are also substituted from secondary care. GP empowerment by training, rewards for additional tasks, and delegation of (other) tasks (for example, to a general practice nurse) may help to solve obstacles.
